# The Effect of Psychological Burden on Dyslipidemia Moderated by Greenness: A Nationwide Study from China

**DOI:** 10.3390/ijerph192114287

**Published:** 2022-11-01

**Authors:** Chengcheng Liu, Yao Li, Jing Li, Chenggang Jin, Deping Zhong

**Affiliations:** 1 School of Social Development and Public Policy, Beijing Normal University, Beijing 100875, China; 2 Faculty of Geographical Science, Beijing Normal University, Beijing 100875, China; 3 National Institute of Natural Hazards, Ministry of Emergency Management of the People’s Republic of China, Beijing 100085, China

**Keywords:** chronic disease, dyslipidemia, depression, NDVI, middle-aged and older Chinese, multilevel mixed-effects logistic regression

## Abstract

Globally, dyslipidemia is now become a leading risk factor for many adverse health outcomes, especially in the middle-aged and elderly. Recent evidence suggests that exposure to greenness and the relief of a psychological burden may decrease the prevalence of dyslipidemia. The objective of our study was to examine whether a green space can moderate the association between mental health status and dyslipidemia. Our study selected the datasets of depression symptoms, dyslipidemia from the China Health and Retirement Longitudinal Study (CHARLS), and the satellite-based normalized difference vegetation index (NDVI) from the 30 m annual maximum NDVI dataset in China in 2018. Ultimately, a total of 10,022 middle-aged and elderly Chinese were involved in our study. Multilevel logistic regressions were performed to examine the association between symptoms of depression and dyslipidemia, as well as the moderate effect of greenness exposure on the association. Our research suggested that adults diagnosed with depression symptoms were more likely to suffer from dyslipidemia. In addition, the NDVI was shown to moderate the effect of depression on dyslipidemia significantly, though the effect was attenuated as depression increased. Regarding the moderate effect of the NDVI on the above association across age, gender, and residence, the findings presented that females, the elderly, and respondents living in urban areas were at a greater risk of having dyslipidemia, although the protective effect of the NDVI was considered. Likewise, the moderate effect of the NDVI gradually decreased as the level of depression increased in different groups. The current study conducted in China provides insights into the association between mental health, green space, and dyslipidemia. Hence, improving mental health and green spaces can be potential targets for medical interventions to decrease the prevalence of dyslipidemia.

## 1. Introduction

Globally, dyslipidemia has posed a major health threat and has been one of the leading causes of death and burden in China [1]. Dyslipidemia is defined as the imbalance of cholesterol, low-density lipoprotein cholesterol, triglycerides, and high-density lipoprotein. It is seen as a key risk factor in the formation and development of cardiovascular disease, stroke heart failure, and renal failure [2,3]. A national survey showed that the overall prevalence of dyslipidemia among Chinese adults is steadily rising [4,5]. Consequently, the economic burden of dyslipidemia among the elder population is also substantial, which attracts much public attention [6].

Although many factors that cause dyslipidemia have been identified (e.g., obesity, nutrition, lack of physical activity, attraction, alcoholism, etc.) [7,8,9,10], the exact causes of dyslipidemia remain unclear [11]. There are two instances of mainstream literature that investigate the causes of dyslipidemia: contextual factors and individual factors. Among the adverse individual factors, recent studies focus on the elevated influence from depression on the formation of dyslipidemia [12,13]. However, it is noteworthy that depression may not have a significant impact on dyslipidemia. The insignificant relation might have been attributed to the definition of dyslipidemia, the method, and the participants [14,15]. Given this background, the relationship between depression and dyslipidemia should be further examined.

Aside from individual factors, accumulating evidence suggests that contextual factors such as socioeconomic factors (e.g., GDP, population distribution, education status, income) might also be related to dyslipidemia [16,17]. Specifically, adults with s high socioeconomic status (SES) might have a lower risk of dyslipidemia than those with a low SES [18]. Other than socioeconomic factors, broadly defined contextual factors, which entail the built environment, water abundance, and greenness exposure, may also exert influence on dyslipidemia [19,20]. Among those, an extensive body of work suggest that adults living in a low-greenness environment were more likely to have a high prevalence of dyslipidemia than their counterparts living in a high-greenness environment [21,22], indicating a beneficial association between greenness and dyslipidemia [23]. In other words, a protective effect of greenness exposure on dyslipidemia has generally been found [24,25,26]. One possible explanation for the effect is that greenness exposure may alleviate the effect of contextual hazards (e.g., air pollution, noise) and enable people to engage in adequate physical activity, as shown in *Exploring Pathways Linking Greenspace to Health: Theoretical and Methodological Guidance* [27], through the improvement of mental health status [28].

**Figure 1:**
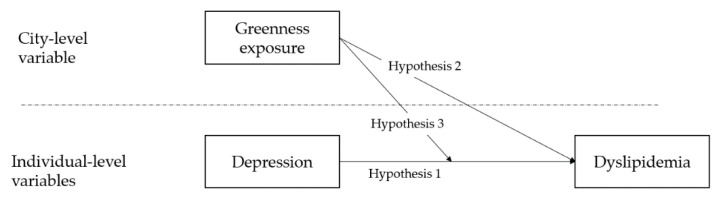
Research hypotheses.

Multiple studies have examined the association between individual/contextual factors and dyslipidemia, but the exact etiology of dyslipidemia remains implicit [29]. To the best of our knowledge, few studies have used both environmental factors and an individual’s psychological attributes—for example, depression—to predict the level of dyslipidemia. Specifically, much less attention has been paid to the moderate effect of greenness on the association between the psychological burden and dyslipidemia, especially in developing countries. In the context of an aging population, the exploration of the relation between greenness, depression, and dyslipidemia may have public health significance in China. Therefore, the objective of our study was to examine whether greenness and depression can be predictive of dyslipidemia. In addition, our study also serves as an attempt to explore the underlying mechanism between greenness, depression, and dyslipidemia, and specifically the moderate effect. Hence, we made three hypotheses (Three hypotheses involved in the study were presented in Figure 1.):

**Hypothesis** **1.** *A higher level of depression is associated with higher dyslipidemia*.

**Hypothesis** **2.** *Greenness exposure can exert influence on an individual’s dyslipidemia*.

**Hypothesis** **3.** *Greenness exposure may function as a moderator that affects the association between depression and dyslipidemia*.

In addition, we also examined the moderate effect of greenness exposure on the association between depression and dyslipidemia across gender, age, and residential place.

## 2. Materials and Methods

### 2.1. Data Collection and Sampling

The datasets for this study were sourced from the China Health and Retirement Longitudinal Study (CHARLS) national survey of wave four in 2018 and the 30 m annual maximum NDVI dataset in China from 2000 to 2020.

**Figure 2:**
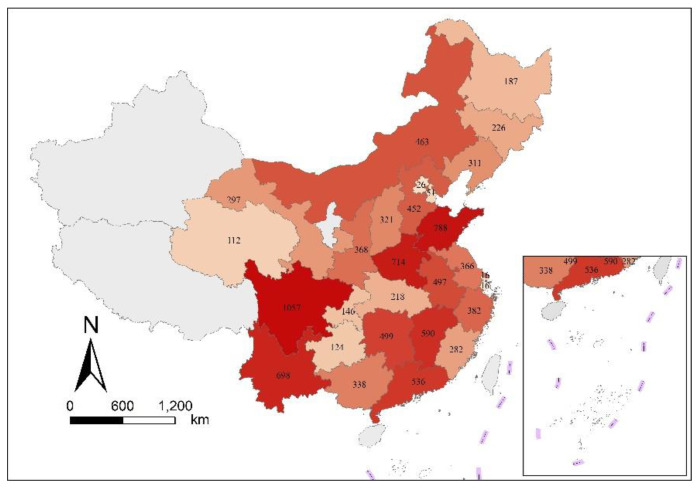
Provincial spatial distribution of valid samples in China.

CHARLS is the first nationally representative survey of Chinese residents aged 45 and older, and has contributed to the research of health of the elderly in China, including studies related to chronic diseases and mental health issues [13,30]. Using the stratified multi-stage PPS random sampling strategy, the national baseline survey of CHARLS was conducted by Peking University in 2011. First, 150 counties/districts were randomly selected from 28 provinces to represent the geographic pattern of all counties in China. Second, 450 urban/villages communities were selected, with each county/district in proportion to the local population size. In the third stage, households in each community unit were mapped and households were then randomly selected. Finally, one resident over 45 years old was selected randomly from each household. Ultimately, the baseline survey of CHARLS covered 28 provinces, 150 counties/districts, and 450 urban/villages communities across the country, involving 17,708 respondents in 10,257 households. The respondents have been followed up every two years. The latest available dataset conducted in 2018 was extracted for our study, involving a total of 19,817 respondents, which could generally reflect the middle-aged and older Chinese population. Moreover, every respondent was interviewed face-to-face, and each interviewer was well trained before conducting field research. More detailed information about CHARLS can be found on its web portal (http://www.charls.pku.edu.cn/en [Accessed on 15 August 2022]). In our study, 10,022 valid samples were included for further analyses since the missing values of the variables in the current study were discarded. Meanwhile, provincial spatial distribution of valid samples was shown in Figure 2.

The 30 m annual maximum NDVI dataset of China in 2018 was conducted by Institute of Geographic Sciences and Natural Resources Research, CAS, which can be retrieved from National Ecosystem Science Data Center, National Science & Technology Infrastructure of China (http://www.nesdc.org.cn [Accessed on 15 August 2022]) [31].

Our study selected the dataset of “demographic backgrounds” section and “Health status and functioning” section from CHARLS, and subsequently merged both datasets with the 30 m annual maximum NDVI dataset in China in 2018. Eventually, the final dataset contained individual-level factors, which entailed demographic characteristics, mental status, chronic diseases, and city-level factors that specifically referred to NDVI.

### 2.2. Instrument

**Dyslipidemia status.** According to the Chinese adult dyslipidemia prevention guide [32] and previous studies [33,34], dyslipidemia contains increased total cholesterol (TC), triglyceride (TG), and low-density lipoprotein cholesterol (LDL-C), and decreased high-density lipoprotein cholesterol (HDL-C). Phenotypes such as TC > 6.2 mmol/L, TG ≥ 2.3 mmol/L, LDL-C ≥ 4.1 mmol/L, and HDL-C < 1.0 mmol/L in male or HDL-C < 1.3 mmol/L in female, or self-reported dyslipidemia, can all be defined as dyslipidemia [13,35]. Similarly, the respondents of CHARLS were asked the question, “Have you been diagnosed with Dyslipidemia (elevation of LDL-C, TGs, and TC, or HDL-C level) by a doctor?”. The item was answered on a scale of “Yes” or “No”.

**Depression symptoms.** The level of respondents’ depression symptoms was screened by the 10-item Center for Epidemiologic Studies Depression (CESD-10) scale, which has been used extensively in China and has exhibited adequate validity and reliability in the elderly [13,36,37]. Participants were asked 10 items about their feelings and behaviors one week prior to the interview, including feeling bothered, having trouble keeping focused, feeling depressed, feeling unable to do anything, feeling hopeless about the future, feeling fearful, having restless sleep, feeling unhappy, feeling lonely, and feeling difficult to get “going”. Each item had the same selective answers and was rated on a 4-point Likert scale: 0 = rarely or none of the time (<1 day), 1 = some (1–2 days), 2 = occasionally (3–4 days), 3 = most or all of the time (5–7 days). The total CESD-10 scores equaled the sum of all items and ranged from 0 to 30, with a higher score suggesting a higher level of depression symptoms. Moreover, the Cronbach’s alpha of CESD-10 in our study was 0.80, indicating acceptable internal consistency [38].

**Normalized Difference Vegetation Index (NDVI).** Due to the high sensitivity to greenness space, NDVI, consisting of vegetation density and vegetation health, is commonly used to estimate and monitor vegetation cover [39,40]. According to studies in the field of epidemiology and geography of health, NDVI has been widely used as the marker of greenness for evaluating its health effect [41,42,43]. NDVI values are bound from −1 to 1 [44]. Generally, a negative value of NDVI or a value close to 0 represents non-biomass with rocks, sand, water, or snow, whereas high positive values indicate healthy greenness [45]. NDVI can be extracted from satellite images [46,47,48], and previous research indicated that the mean values are used to examine the effect of neighborhood green space on individual health directly [46,49,50]. Therefore, to determine city-level NDVI level, ArcGIS was used to extract and calculate the average of all raster data within certain city.

**Potential confounding variables.** In light of existing studies of dyslipidemia [13,51], gender (male, female), residence (central of city/town, urban–rural integration zone, rural), marital status (unmarried, married, separated/divorced/widowed), smoking (smoking, non-smoking), alcohol use one year prior to the interview (drink more than once a month, drink but less than once a month, none of these), age, years of education, and self-reported health status (very poor, poor, fair, good, very good) were included in covariates.

### 2.3. Data Analysis

**Figure 3:**
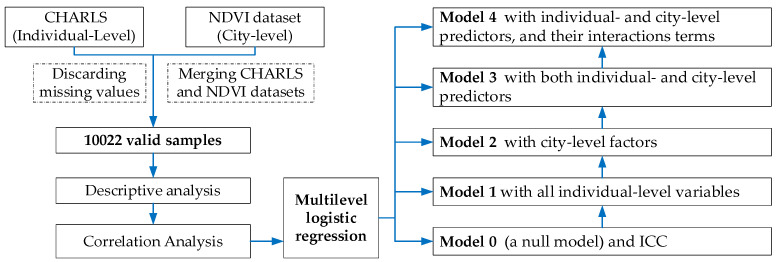
Flowchart of the study.

Data analyses were conducted using STATA. Descriptive statistic was used to summarize the frequency and percentages or the mean values of characteristics of study participants and city-level NDVI. The correlation matrix model was performed to identify the relationship between dyslipidemia and individual characteristics, as well as city-level NDVI. Furthermore, due to the hierarchical structure of dataset and the binary outcome variable of dyslipidemia, multilevel mixed-effects logistic regression was fitted to identify the predictors of dyslipidemia in China, which has been increasingly recognized as a useful tool for examining the effect from group level and individual level simultaneously [52]. Flowchart of the study were illustrated in Figure 3. 

#### 2.3.1. Multilevel Logistic Regression Description

For multilevel logistic regression with two levels (individual level and group level), the model equations can be presented as follows:

logit (π_ij_) = β_0j_ + β_1_depression_ij_ + β_2_gender_ij_ + β_3_residence_ij_ + β_4_marital_ij_ + β_5_smoking_ij_ + β_6_alcohol_ij_ + β_7_age_ij_ + β_8_education_ij_ + β_9_health_ij_ (1)

where $\pi_{\mathrm{ij}}$ presents the probability of suffering for individual  i nested in county j, $\beta_{0j}$ is the city-level intercept, and $\beta_{1}$ is the regression coefficient corresponding to the effect of individual-level factors such as depression, gender, residence, marital status, smoking, alcohol use, age, years of education, and self-reported health status.

β_0j_ = γ_00_ + γ_01_NDVI_j_ + δ_0j_ (2)

The city-level intercept defined in Equation (1) can be shown in detail in Equation (2), where γ_01_ is the regression coefficient corresponding to the effect of city-level NDVI, and δ_0j_ is the independent and identically distributed error term.

#### 2.3.2. Model Building and Measure

In the current study, five multilevel logistic regression models were carried out:

(1) A null model (Model 0) without any predictors was run first and corresponding intraclass correlation coefficient (ICC) was also calculated [53]. Here, ICC refers to the amount of variance in individual level response that can be explained by city-level properties [54]. In general, ICC greater than 0.059 suggests that multilevel regression is acceptable [55,56].
(2) Model 1 included all individual-level variables to ascertain their association with dyslipidemia.
(3) Model 2 used city-level factors to predict the effect of city-level NDVI on dyslipidemia.
(4) All individual- and city-level predictors were then involved in Model 3 (the full model).
(5) According to the results of Model 3, Model 4 was subsequently constructed with depression status, NDVI, and their interaction terms to examine the moderating effect of city-level NDVI on the depression–dyslipidemia relationship.

A likelihood ratio test was performed after each multilevel logistic regression to determine whether two-level model was more appropriate than individual-level model [57,58]. In addition, Akaike’s information criterion (AIC) and Bayesian information criterion (BIC) were utilized to check fitness of different models [59]. Odd ratios (ORs) and corresponding 95% confidence intervals (95% CIs) were used to estimate the association between dyslipidemia and its potential predictors. An OR value greater than 1.00 indicated that predictor could increase the likelihood of dyslipidemia [60]. The significance level was set at *p* value < 0.05.

## 3. Results

### 3.1. Descriptive Analysis

Table 1 illustrates descriptive statistics for individual-level characteristics and the city-level NDVI. Overall, 13.13% of the respondents reported that they had dyslipidemia. The age ranged from 45 to 98 (M = 63.01, SD = 9.86), almost 59% of them were female, and 83.71% were married. More than 75% of participants lived in rural areas, and 23.77% were in the center of a city/town or urban–rural integration zone. Moreover, the proportion of smoking was 24.43%, and distributions for alcohol use were similar. On average, the participants had at least 5 years of education. Moreover, the mean depression symptoms (±SD) were 9.67 ± 6.66. Regarding the NDVI level, the average value was 0.77, and the standard deviation was 0.08. More details are shown in Table 1.

### 3.2. Correlation Analysis

Table 2 summarizes results from the correlation analysis, indicating that dyslipidemia was significantly associated with depression, NDVI, residence, smoking status, alcohol use, age, years of education, and self-reported health status. In detail, those with higher depression symptoms were more likely to suffer from dyslipidemia. Regarding the city-level NDVI, the findings suggested that it was a protective predictor for dyslipidemia. In addition, depression symptoms were found to be significantly correlated with gender, residence, marital status, smoking status, alcohol use, age, years of education, and self-reported health status. Moreover, depression symptoms were significantly different regarding the city-level NDVI. The details are provided in Table 2.

### 3.3. Multilevel Logistic Regression

**Figure 4:**
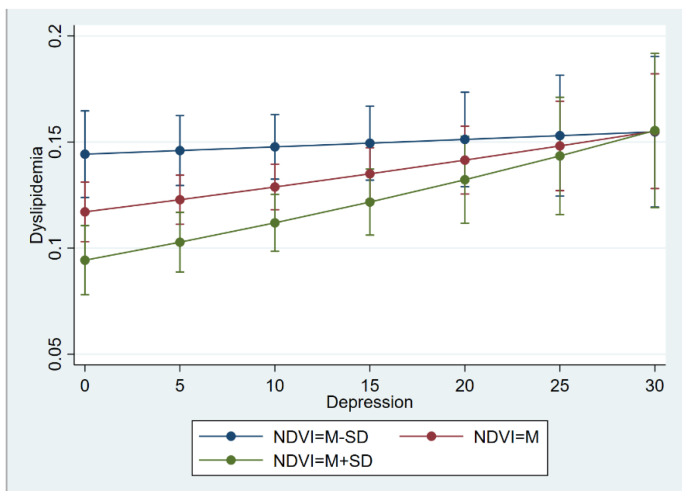
The interaction effect of depression and NDVI on dyslipidemia.

The findings of the multilevel logistic regression models are displayed in Table 3, which were constructed to predict the association between dyslipidemia, depression, NDVI, and other potential predictors. For Model 0 with ICC estimates, the ICC estimates (ICC = 0.068 > 0.059) indicated substantial variation at the city level, which supported the multilevel specification. Further, Model 1 showed that depression was a risk predictor of dyslipidemia (OR = 1.01, 95% CI: 1.00–1.02), and that respondents who suffered from depression were 1.01 times more likely to have dyslipidemia than those without mental health issues. In addition, the city-level NDVI was confirmed to be associated with dyslipidemia at the individual level. Specifically, respondents who lived with a higher NDVI were less likely to develop dyslipidemia disease (OR = 0.12, 95% CI: 0.04–0.37) than those with a lower NDVI. The results are shown in Model 2 of Table 3. When including both individual-level depression and the city-level NDVI, the results of Model 3 suggested that individual depression (OR = 1.01, 95% CI: 1.00–1.02) and the city NDVI (OR = 0.11, 95% CI: 0.04–0.36) were related to dyslipidemia significantly, respectively. The moderate effect of the NDVI on the relationship between depression and dyslipidemia was also estimated. The results reveled that the effect of depression on dyslipidemia was moderated by the NDVI (OR = 1.12, 95% CI: 1.00–1.24), which is listed in Model 4 and Figure 4. In Figure 4, there are three lines indicating the influence of the three levels of the NDVI on the association between depression and dyslipidemia. The higher level of the NDVI (green line) makes more of an effort to reduce the level of dyslipidemia compared with the medium level of the NDVI (red line) and low level of the NDVI (blue line). For example, if the individuals’ depression level is 0, those who have a higher level of the NDVI may suffer less from dyslipidemia. However, the effect of the NDVI was limited. Specifically, the moderate effect of the NDVI was attenuated when the level of depression approached 30. In other words, the NDVI has a very limited effect on those who have severe depression. Moreover, the values of the log likelihood, AIC, and BIC presented in Table 3 illustrated that there were obvious improvements in the models described previously.

Further, multilevel logistic regression was also performed to test the moderate effect of the NDVI on the relationship between depression and dyslipidemia across groups such as age (45–59/60+), gender (male/female), and residence (central of city/town, urban–rural integration zone, rural).

#### 3.3.1. The Moderate Effect across Age Groups

**Figure 5:**
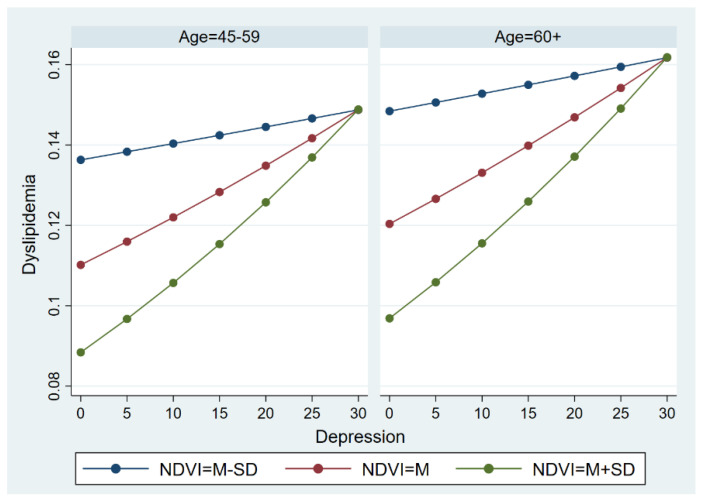
The moderate effect of NDVI on the relationship between depression and dyslipidemia across age groups.

We sought to determine whether the moderate effect of the NDVI on the relationship between depression and dyslipidemia was variant in the younger group (respondents aged 45–59) and the elderly (Figure 5). In general, the NDVI functioned as a moderator that affects the association between depression and dyslipidemia. Similar to the results among the whole sample, the effect of the NDVI was attenuated when the level of depression approached 30. In comparison, the elderly were more likely to suffer from dyslipidemia than those aged 45–59, indicating that there were obvious differences in the model in younger subjects and the elderly.

#### 3.3.2. The Moderate Effect across Gender Groups

**Figure 6:**
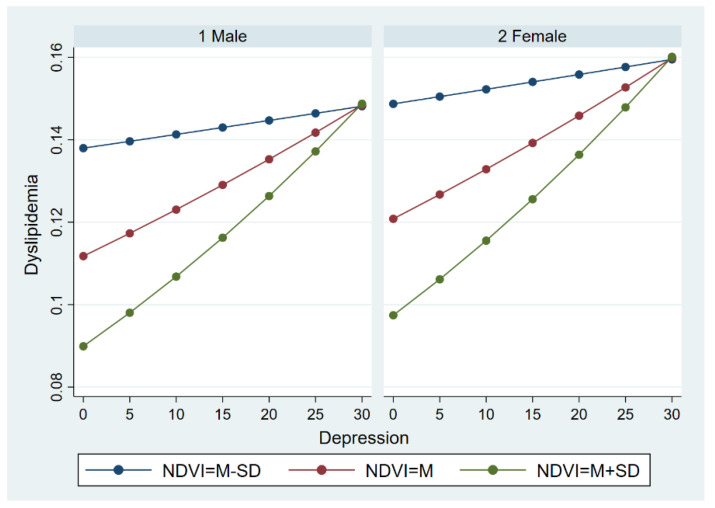
The moderate effect of NDVI on the relationship between depression and dyslipidemia across gender groups.

Multilevel logistic regression was further carried out to examine the moderate effect described previously across gender groups. Similar to the results among the whole sample, the effect of depression on dyslipidemia can also be moderated by the NDVI, both in the male and female group. Likewise, the moderate effect of the NDVI gradually decreased as the level of depression increased. However, there was an apparent difference in dyslipidemia between the male group and female group. For a given depression and a given NDVI level, females were at a greater risk of dyslipidemia than males. The details are illustrated in Figure 6.

#### 3.3.3. The Moderate Effect across Residence Groups

**Figure 7:**
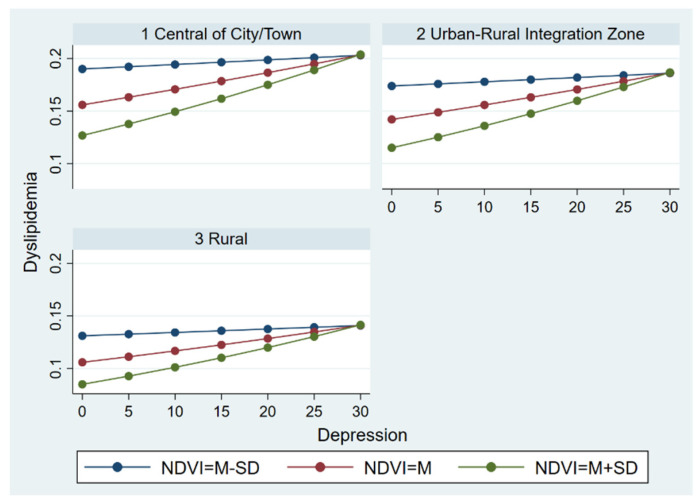
The moderate effect of NDVI on the relationship between depression and dyslipidemia across residence groups.

Additionally, we conducted multilevel logistic regression to determine if the test model was statistically different across residence groups. Figure 7 seems to indicate that there were residence differences in the moderate effect of the NDVI on the relationship between depression and dyslipidemia. Specifically, symptoms of depression were a greater risk factor for dyslipidemia in respondents living in urban areas than those living in rural areas and the urban–rural integration zone, though the protective effect of the NDVI was considered. Correspondingly, those living in rural areas and the urban–rural integration zone were less likely to be diagnosed with dyslipidemia for a given depression and a given NDVI level. Moreover, the results from three groups simultaneously exhibited that the protective effect of the NDVI continued to diminish as self-reported symptoms of depression increased.

## 4. Discussion

The objective of this study was to investigate factors associated with dyslipidemia among a national representative sample (CHARLS and the 30 m annual maximum NDVI dataset of China) of 10,022 elder people. Specifically, multilevel logistic regression was employed to examine the association between depression and dyslipidemia and the association between greenness exposure and dyslipidemia. We also examined the moderate effect of greenness exposure on the association between depression and dyslipidemia across gender, age, and residential place.

Our findings suggested that depression was associated with dyslipidemia, and thus Hypothesis 1 was supported. This is consistent with previous work showing that depression can be a leading risk factor for negative life attitude [61], lifestyle habit (e.g., smoking, drinking) [62,63], incremental nutritional imbalance, and less physical activity engagement [64]. For example, those who suffer from depression were more likely to smoke in order to modulate stress. However, smoking behavior reduced HDL cholesterol and thus increased the level of dyslipidemia [65,66].

A protective effect of greenness exposure on dyslipidemia has also been found. The Hypothesis 2, that exposure to greenness is associated with dyslipidemia, was supported and is consistent with previous work [20,21,67]. Although our study estimated the association between greenness exposure and dyslipidemia at city level, our findings may also support those studies that focused on the community level [20,21,67]. For example, in a previous study of a Chinese elderly population, Shujun Fan reported that those who were surrounded by more greenness may have a lower level of TG and higher level of HDL-C, indicating a beneficial association between greenness exposure and dyslipidemia [20]. One possible explanation is that greenness can reduce air pollution levels and thus contribute to people’s health condition [25]. Another explanation can be that greenness exposure may promote opportunities for more physical activity engagement [68]. However, greenness exposure may not have a significant impact on dyslipidemia across a diversified greenness area, vegetation type, and exposure level [69]. One way to interpret the result is to speculate that the time that people are exposed to greenness may function as confounding bias in the relationship between greenness exposure and dyslipidemia [70].

Our findings also suggested that greenness exposure functions as a moderator to the association between depression and dyslipidemia, indicating that the greater exposure to greenness may lessen the effect of depression on dyslipidemia. This is also consistent with prior work that showed that greenness exposure can moderate the association between depression and other chronic diseases (e.g., hypertension) [71,72,73]. The potential underlying mechanism could be that greenness may reduce the negative impact from environmental stressors (e.g., heat and noise) to people, and, thus, the decreasing psychological burden may reduce the level of dyslipidemia [74]. Hence, Hypothesis 3, that the greenness exposure can moderate the association between depression and dyslipidemia, was confirmed. However, it is also worth noting that the incremental depression hampered the moderate effect of greenness exposure on the depression–dyslipidemia relationship. One way to interpret our finding is to speculate that the increasing mental illness may be the cause of lifestyle habits (e.g., smoking and drinking), and those lifestyle habits may hamper people engaging in physical activity, even under the beneficial influence of greenness exposure [64].

In addition, we also examined the moderate effect of greenness exposure on the depression–dyslipidemia relationship across groups. Our results generally indicated that the moderate effect of greenness exposure on the association between depression dyslipidemia could vary by age, gender, and urban–rural difference. With regard to the age group, the moderate effect on the association was contingent on the age. Compared with age group (45–59 years old), dyslipidemia of those who are aged more than 60 years old is more likely to be affected by depression, even when highly exposed to greenness [75]. This result is congruent with prior studies that suggested that age was associated with the level of dyslipidemia [29,76]. With regard to the gender difference, our result suggested that female adults were more likely to have a higher level of dyslipidemia than the male [15]. This generally agrees with previous studies that showed that the underlying biological mechanisms for the association between gender and dyslipidemia could be attributed to the female’s increasing progesterone [77,78]. In addition, the moderate effect of greenness exposure on the association between depression and dyslipidemia was also modified by the urban–rural difference due to the high density of the urban area [77].

Furthermore, our study is consistent with prior studies suggesting associations between covariates and dyslipidemia, such as gender [79], urban–rural difference [77], marital status [80], age [29], years of education [81], smoking status [82], and alcohol use [83,84].

Overall, our study has several strengths. First, we adopted CHARLS as our primary dataset since it was a high-quality (nationally representative) dataset and used widely by scholars from diversified realms, such as economic and public policy. Second, to the best of our knowledge, our study is the first one to examine the moderate effect of greenness on the association between depression and dyslipidemia. Third, we used two-level multilevel logistic regression to examine the effect from both context-level and individual-level predictors.

## 5. Conclusions

In conclusion, we employed multilevel logistic regression to examine the association between greenness exposure, depression, and dyslipidemia by using data from CHARLS and the NDVI. Evidence suggested that exposure to greenness can moderate the association between depression and dyslipidemia. Our findings also shed light on the moderate effect across different genders and age groups. Our study provides evidence for coping with dyslipidemia from an integrated context-individual perspective.

Some limitations of this study should be acknowledged. First, a causal relationship between greenness exposure, depression, and dyslipidemia cannot be deduced due to cross-sectional data used in our study. Second, we mainly focused on the relationship between greenness exposure, depression, and dyslipidemia, and confounding bias other than marital status, age, and years of education should be further involved. Third, we measured greenness exposure by calculating a static NDVI instead of a dynamic one. In future studies, more remote sensing data, such as the enhanced vegetation index (EVI), should be used to increase the accuracy and precision of study outcomes.

## Figures and Tables

**Table 1:** Descriptive analysis (N = 10,022).

Variable	Frequency	Percent (%)
Dyslipidemia		
No	8706	86.87
Yes	1316	13.13
Gender		
Male	4137	41.28
Female	5885	58.72
Residence		
Central of city/town	1691	16.87
Urban–rural integration zone	692	6.90
Rural	7639	76.22
Marital status		
Unmarried	57	0.57
Married	8389	83.71
Separated/divorced/widowed	1576	15.73
Smoking		
Smoking	2448	24.43
Non-smoking	7574	75.57
Alcohol use		
Drink more than once a month	2277	22.72
Drink but less than once a month	688	6.86
None of these	7057	70.42
	Mean	Standard Deviation
Depression symptoms	9.67	6.66
NDVI	0.77	0.08
Age	63.01	9.86
Years of education	5.52	3.82
Self-reported health status	2.85	0.96

**Table 2:** Correlation analysis (N = 10,022).

	X1	X2	X3	X4	X5	X6	X7	X8	X9	X10	X11
**X1**	1.00										
**X2**	0.04 ***	1.00									
**X3**	−0.05 ***	0.04 ***	1.00								
**X4**	0.02	0.12 ***	−0.01	1.00							
**X5**	−0.08 ***	0.13 ***	−0.04 ***	−0.03 *	1.00						
**X6**	−0.02	0.07 ***	0.02	0.13 ***	−0.02 *	1.00					
**X7**	0.03 **	0.04 ***	−0.03 **	0.54 ***	−0.05 ***	0.06 ***	1.00				
**X8**	0.02 *	0.09 ***	−0.02	0.43 ***	0.01	0.08 ***	0.32 ***	1.00			
**X9**	−0.02 *	−0.04 ***	0.04 ***	−0.10 ***	0.01	0.31 ***	−0.00	0.04 ***	1.00		
**X10**	0.07 ***	−0.14 ***	−0.01	−0.28 ***	−0.29 ***	−0.14 ***	−0.12 ***	−0.17 ***	−0.23 ***	1.00	
**X11**	−0.11 ***	−0.34 ***	−0.05 ***	0.00	−0.08 ***	−0.03 **	−0.01	−0.08 ***	−0.05 ***	0.07 ***	1.00

Note: X1 = dyslipidemia, X2 = depression, X3 = NDVI, X4 = gender, X5 = residence, X6 = marital status, X7 = smoking, X8 = alcohol use, X9 = age, X10 = years of education, X11 = self-reported health status. *** means *p* < 0.001, ** means *p* < 0.01, * means *p* < 0.05.

**Table 3:** Multilevel logistic regression (N = 10,022).

Variable	Model 0 OR (95% CI)	Model 1 OR (95% CI)	Model 2 OR (95% CI)	Model 3 OR (95% CI)	Model 4 OR (95% CI)
**Intercept**	0.14 (0.13–0.16) ***	0.34 (0.12–0.93) *	2.14 (0.57–8.01)	1.78 (0.47–6.77)	4.14 (0.87–19.83)
**NDVI**	---	---	0.12 (0.04–0.37) ***	0.11 (0.04–0.36) ***	0.04 (0.01–0.18) ***
**Depression symptoms**	---	1.01 (1.00–1.02) *	---	1.01 (1.00–1.02) *	0.93 (0.85–1.01)
NDVI * Depression symptoms	---	---	---	---	1.12 (1.00–1.24) *
**Gender** (Ref: male)					
Female	---	1.09 (0.93–1.29)	1.11 (0.95–1.31)	1.10 (0.93–1.29)	1.10 (0.93–1.29)
**Residence** (Ref: central of city/town)					
Urban–rural integration zone	---	0.90 (0.71–1.16)	0.90 (0.70–1.15)	0.90 (0.70–1.15)	0.89 (0.70–1.14)
Rural	---	0.64 (0.54–0.76) ***	0.64 (0.54–0.76) ***	0.63 (0.54–0.75) ***	0.63 (0.53–0.74) ***
**Marital status** (Ref: unmarried)					
Married	---	1.22 (0.53–2.79)	1.20 (0.52–2.73)	1.22 (0.53–2.78)	1.21 (0.53–2.77)
Separated/divorced/widowed	---	1.04 (0.45–2.42)	1.04 (0.45–2.40)	1.04 (0.45–2.41)	1.04 (0.45–2.41)
**Age**	---	1.00 (0.99–1.00)	1.00 (0.99–1.00)	1.00 (0.99–1.01)	1.00 (0.99–1.00)
**Years of education**	---	1.04 (1.02–1.06) ***	1.04 (1.02–1.06) ***	1.04 (1.03–1.06) ***	1.04 (1.02–1.06) ***
**Smoking status** (Ref: smoking)					
Non-smoking	---	1.14 (0.96–1.35)	1.13 (0.95–1.34)	1.13 (0.95–1.35)	1.13 (0.95–1.35)
**Alcohol use** (Ref: drink more than once a month)					
Drink but less than once a month	---	1.13 (0.87–1.46)	1.13 (0.87–1.47)	1.12 (0.86–1.46)	1.12 (0.86–1.45)
None of these	---	1.08 (0.92–1.28)	1.09 (0.92–1.29)	1.08 (0.91–1.28)	1.08 (0.91–1.28)
**Self-reported health status**	---	0.66 (0.61–0.71) ***	0.64 (0.60–0.69) ***	0.66 (0.61–0.71) ***	0.66 (0.61–0.71) ***
**Log Likelihood**	−3844.981	−3724.783	−3720.7126	−3718.382	−3716.371
**AIC**	7693.962	7477.566	7469.425	7466.764	7464.742
**BIC**	7708.387	7578.541	7570.401	7574.952	7580.143

Note: *** means *p* < 0.001, * means *p* < 0.05.

## Data Availability

Dataset is provided by the National Ecosystem Science Data Center, National Science & Technology Infrastructure of China (http://www.nesdc.org.cn [Accessed on 15 August 2022]).
